# Natural tannin extracts supplementation for COVID-19 patients (TanCOVID): a structured summary of a study protocol for a randomized controlled trial

**DOI:** 10.1186/s13063-021-05281-x

**Published:** 2021-04-28

**Authors:** Silvia Molino, Andrea Pisarevsky, Fabiana Lopez Mingorance, Patricia Vega, Juan Pablo Stefanolo, Julieta Repetti, Guillermina Ludueña, Pablo Pepa, Juan Ignacio Olmos, Marcelo Rodriguez Fermepin, Tatiana Uehara, Sonia Villapol, Tor Savidge, Todd Treangen, Elisa Viciani, Andrea Castagnetti, Maria Marta Piskorz

**Affiliations:** 1 Departamento de Nutrición y Bromatología, Instituto de Nutrición y Tecnología de Alimentos, Centro de Investigación Biomédica, Universidad de Granada, Granada, Spain; 2 Departamento de Medicina Interna, Hospital de Clinicas José de San Martin Universidad de Buenos Aires, Buenos Aires, Argentina; 3 Programa de Estudios Pancreáticos, Hospital de Clinicas José de San Martin Universidad de Buenos Aires / IBIMOL, Buenos Aires, Argentina; 4 Hospital de Gastroenterologia Dr Carlos Bonorino Udaondo, Buenos Aires, Federal District Argentina; 5 Servicio de Gastroenterología. Hospital de Clinicas Jose de San Martin, Universidad de Buenos Aires, Buenos Aires, Argentina; 6 Hospital de Clinicas Jose de San Martin Universidad de Buenos Aires, Buenos Aires, Argentina; 7 Departamento de Bioquímica Clínica, Facultad de Farmacia y Bioquímica, Universidad de Buenos Aires, Hospital de Clinicas José de San Martin, Buenos Aires, Argentina; 8 Sector Neurogastroenterología, Hospital de Clinicas José de San Martin Universidad de Buenos Aires, Buenos Aires, Argentina; 9 Houston Methodist Research Institute, Houston, TX United States; 10 Baylor College of Medicine, Houston, TX United States; 11 Rice University Department of Computer Science, Houston, TX United States; 12 Wellmicro, Bologna, Italy

**Keywords:** COVID-19, Randomized controlled trial, protocol, Quebracho tannins, Chestnut tannins, gut microbiota, inflammation

## Abstract

**Objectives:**

This research aims to study the efficacy of tannins co-supplementation on disease duration, severity and clinical symptoms, microbiota composition and inflammatory mediators in SARS-CoV2 patients.

**Trial design:**

This is a prospective, double-blind, randomized, placebo-controlled, parallel-group trial to evaluate the efficacy of the administration of the dietary supplement ARBOX, a molecular blend of quebracho and chestnut tannins extract and Vit B12, in patients affected by COVID-19.

**Participants:**

18 years of age or older, admitted to Hospital de Clinicas Jose de San Martin, Buenos Aires University (Argentina), meeting the definition of "COVID-19 confirmed case" (https://www.argentina.gob.ar/salud/coronavirus-COVID-19/definicion-de-caso).

*Inclusion Criteria*

Participants are eligible to be included in the study if the following criteria apply:

1. Any gender
2. ≥18 years old
3. Informed consent for participation in the study
4. Virological diagnosis of SARS-CoV-2 infection (real-time PCR)

*Exclusion Criteria*

Participants are excluded from the study if any of the following criteria apply:

1. Pregnant and lactating patients
2. Patients who cannot take oral therapy (with severe cognitive decline, assisted ventilation, or impaired consciousness)
3. Hypersensitivity to polyphenols
4. Patients already in ICU or requiring mechanical ventilation
5. Patients already enrolled in other clinical trials
6. Decline of consent

**Intervention and comparator:**

Experimental: *TREATED ARM*

Participants will receive a supply of 28 -- 390 mg ARBOX capsules for 14 days. Patients will be supplemented with 2 capsules of ARBOX per day.

Placebo Comparator: *CONTROL ARM*

Participants will receive placebo supply for 14 days. The placebo will be administered with the identical dose as described for the test product.

All trial participants will receive standard therapy, which includes: Antipyretics or Lopinavir / Ritonavir, Azithromycin and Hydroxychloroquine, as appropriate (treatment currently recommended by the department of Infectious Diseases of the Hospital de Clínicas that could undergo to modifications). In addition, if necessary: supplemental O2, non-invasive ventilation, antibiotic therapy.

**Main outcomes:**

*Primary Outcome Measures*:

Time to hospital discharge, defined as the time from first dose of ARBOX to hospital discharge [ Time Frame: Throughout the Study (Day 0 to Day 28) ]

*Secondary Outcome Measures:*

- 28-day all-cause mortality [ Time Frame: Throughout the Study (Day 0 to Day 28) ]-proportion
- Invasive ventilation on day 28 [ Time Frame: Throughout the Study (Day 0 to Day 28) ]-proportion
- Level of inflammation parameters and cytokines [ Time Frame: day 1-14 ] -mean difference
- Difference in fecal intestinal microbiota composition and intestinal permeability [ Time Frame: day 1-14 ]
- Negativization of COVID-PCR at day 14 [ Time Frame: day 14 ]-proportion

**Randomization:**

Potential study participants were screened for eligibility 24 hours prior to study randomization. Patients were randomly assigned via computer-generated random numbering (1:1) to receive standard treatment coupled with tannin or standard treatment plus placebo (control group).

**Blinding (masking):**

Study personnel and participants are blinded to the treatment allocation, as both ARBOX and placebo were packed in identical containers. Thus, all the used capsules had identical appearance.

**Numbers to be randomized (sample size):**

Considering an alpha error of 5%, a power of 80% a sample size of 70 patients per branch was estimated. 140 patients in total.

**Trial Status:**

The protocol version is number V2, dated May 23, 2020.

The first patient, first visit was on June 12, 2020; the recruitment end date was October 6, 2020.

The protocol was not submitted earlier because the enrollment of some patients took place after the closure of the recruitment on the clinicaltrials platform. In fact, due to the epidemiological conditions, due to the decrease of the cases in Argentina during the summer period, the recruitment stopped t before reaching the number of 140 patients (as indicated in the webpage). However, since there was a new increase in cases, the enrolment was resumed in order to reach the number of patients initially planned in the protocol. The final participant was recruited on February 14, 2021.

**Trial registration:**

ClinicalTrials.gov, number: NCT04403646, registered on May 27th, 2020.

**Full protocol:**

The full protocol is attached as an additional file, accessible from the Trials website (Additional file 1). In the interest in expediting dissemination of this material, the familiar formatting has been eliminated; this Letter serves as a summary of the key elements of the full protocol.

**Supplementary Information:**

The online version contains supplementary material available at 10.1186/s13063-021-05281-x.

## Supplementary Information

**Additional file 1.** Full study protocol.

## Data Availability

Not applicable

