# Supplementary material for: Natural tannin extracts supplementation for COVID-19 patients (TanCOVID): a structured summary of a study protocol for a randomized controlled trial
Source: Trials. 2021 Apr 28;22:310. doi: 10.1186/s13063-021-05281-x (PMC8080998; doi:10.1186/s13063-021-05281-x)
Supplement: Supplementary file 1 — Additional file 1. Full study protocol. [file 13063_2021_5281_MOESM1_ESM.docx]

***CLINICAL TRIAL PROTOCOL***

***Study Title:***

**NATURAL TANNIN EXTRACTS FOR COVID-19: A RANDOMIZED CONTROLLED CLINICAL TRIAL.**

**Short title: NATURAL TANNIN EXTRACTS FOR COVID-19**

**Protocol code: TanCOVID**

**ClinicalTrials.gov Identifier: NCT04403646**

**Version | Date: 2. 23/05/2020**

**Sponsor: INDUNOR/ SILVATEAM SA**

**Names protocol contributors**

Andrea Pisarevsky^1^, Silvia Molino^2^, Fabiana Lopez Mingorance^3^, Patricia Vega^1^, Juan Pablo Stefanolo^4^, Julieta Repetti^1^, Guillermina Ludueña^1^, Pablo Pepa^1^, Juan Ignacio Olmos^1^, Marcelo Rodriguez Fermepin^1^, Tatiana Uehara^1^, Sonia Villapol^5^, Tor Savidge^6^, Todd Treangen^7^, Elisa Viciani^8^, Andrea Castagnetti^8^, Maria Marta Piskorz^1^

**Abstract**

**Background**:

There is evidence that the gut microbiota and its relationship with the immune system could be involved in the pathogenesis of COVID-19. SARS-CoV-2 can cause gastrointestinal symptoms during the early phases of the disease. Intestinal dysbiosis induces changes in gut microbiota composition and function, and an increase in inflammatory cytokines. Therefore, microbiota modulation could play a role in COVID-19 treatment. Tannins have been shown to work as prebiotics. In particular, quebracho and chestnut tannins have shown to regulate the immune response and decrease cytokines production, inducing the microbiota to produce secondary metabolites, such as quercetin and SCFAs.

**Methods**: This prospective, double-blind, and randomized study was approved by the Hospital de Clínicas, José de San Martín (Buenos Aires, Argentina). Blood and stool samples will be collected at baseline (Day 0) and after treatment (Day 14) during July-October 2020, with final follow-up in November 2020. We randomly assigned 124 RT-PCR confirmed COVID-19 cases (>18 years) to receive the dietary supplement ARBOX (containing dry extracts of quebracho and chestnut tannins (240 mg) and B12 vitamin (0.72 µg)) or placebo, twice daily for 14 days as adjunct treatment to their standard of care management. A panel of 27- pro and anti-inflammatory cytokines will be measured on day 0 and 14 (Bio-Plex Pro™, Bio- Rad). Final enrollment of 140 patients with matched fecal microbiome characterization (16S, WGS and metabolites) is expected.

**Discussion**: The available information suggests that the antiviral, anti-inflammatory and antioxidant activities of tannins could be a strategy to prevent and control viral respiratory diseases, as well as to diminish the more lethal effects such as excess inflammatory response. Furthermore, oxidative stress and stress-mediated complications (such as inflammation) of viral infections can be successfully countered with antioxidant prevention. However, it has to be highlighted that the application of tannins in the prevention and treatment of viral infections has an auxiliary function and may be particularly beneficial when used in combination with a specific therapy.

**Trial registration**: NCT number: NCT04403646, registered on May 27th, 2020.

**Keywords**

COVID-19, Randomized controlled trial, protocol, Quebracho tannins, Chestnut tannins, gut microbiota, inflammation

**Administrative information**

| Title {1} | NATURAL TANNIN EXTRACTS SUPPLEMENTATION FOR COVID-19 (TanCOVID): A RANDOMIZED CONTROLLED CLINICAL TRIAL. |
| --- | --- |
| Trial registration {2a and 2b}. | NCT number: NCT04403646, registered on May 27th, 2020. |
| Protocol version {3} | V2 23/05/2020 |
| Funding {4} | INDUNOR/ SILVATEAM SA |
| Author details {5a} | 1. HOSPITAL DE CLINICAS JOSE DE SAN MARTIN, BUENOS AIRES, ARGENTINA 2. DEPARTAMENTO DE NUTRICIÓN Y BROMATOLOGÍA, INSTITUTO DE NUTRICIÓN Y TECNOLOGÍA DE ALIMENTOS, CENTRO DE INVESTIGACIÓN BIOMÉDICA, UNIVERSIDAD DE GRANADA, GRANADA, SPAIN. 3. HOSPITAL DE CLINICAS JOSE DE SAN MARTIN/ IBIMOL, BUENOS AIRES, ARGENTINA. 4. HOSPITAL DE GASTROENTEROLOGIA DR CARLOS BONORINO UDAONDO, BUENOS AIRES, FEDERAL DISTRICT, ARGENTINA. 5. HOUSTON METHODIST RESEARCH INSTITUTE, HOUSTON, TX, UNITED STATES. 6. BAYLOR COLLEGE OF MEDICINE, HOUSTON, TX, UNITED STATES. 7. RICE UNIVERSITY DEPARTMENT OF COMPUTER SCIENCE, HOUSTON, TX, UNITED STATES. 8. WELLMICRO, BOLOGNA, ITALY. |
| Name and contact information for the trial sponsor {5b} | Daniela Allerbon  [dallerbon@silvateam.com](mailto:dallerbon@silvateam.com)  +54 91158465136 |
| Role of sponsor {5c} | Founding |

**Introduction**

**Background and rationale {6a}**

**RATIONALE**

Over the past two decades, new coronavirus infections have periodically emerged in several countries, around the world. Severe acute respiratory syndrome coronavirus (SARS-CoV) broke out in 2002, infecting 8422 people and causing 916 deaths during the epidemic. Middle East Syndrome Coronavirus (MERS-CoV) was first identified in 2012: a total of 2499 cases were laboratory-confirmed. In December 2019 a new coronavirus, named SARS-Cov-2, has caused a global respiratory disease called Covid-19. The full spectrum of the disease ranges from mild, self-limiting respiratory symptoms to severe pneumonia, multi-organ failure and death. So far there is no specific treatment for this disease.

In Argentina as of April 3^rd^ there are more than 1200 cases and 37 deaths due to the disease and it is estimated that there are around 250,000 cases. Therefore, there is an urgent need for effective intervention to treat symptomatic patients, reduce the duration of virus carriage and limit transmission in the community.

Several agents that may be effective against SARS-Cov-2 are currently being studied. Natural antiviral agents may be of particular interest because they are widely available and can be used as part of the diet to counteract diseases, including viral infections. In addition, the use of antioxidant compounds helps to negatively regulate macrophage activation and pro-inflammatory cytokines in respiratory cells.

Polyphenols comprise a large group of natural compounds of botanical origin, recognized for their beneficial health effects in various disease models.[1] These compounds are present in foods in the human diet and can also be found in supplements as pure extracts. Among them, tannins have been shown to have antioxidant, anti-inflammatory, antimicrobial and gastrointestinal metabolism regulating properties. As a result, tannins are now widely used in various countries, such as those of the European Union, in both veterinary and human medicine.[2] Available data from numerous in vitro and in vivo trials indicate strong support for the use of tannins for the prevention and control of viral diseases of the respiratory system, including severe symptoms and lethality.[3] The positive action is based on the combination of strong antioxidant power, anti-inflammatory action, metabolic enhancement of epithelial cells and direct antiviral action. Furthermore, it is noteworthy that the use of these compounds (tannins) is a low-cost strategy for prevention and as a complement to standard treatment, for the individual and for public health.

**BACKGROUND**

**Tannins: biological effects.**

Tannins are secondary metabolites, produced by plants as a defence against external agents. These compounds are present in all parts of the plant: root, stem, leaf, fruit and seed. Historically, tannins have been used, as water-soluble polyphenols, in medicines for the treatment of various health problems. In fact, most of the biological effects of these phenolic compounds are of medical importance.[4]

The antioxidant effect protects the cells of the human body against free radicals and enables the prevention of degenerative diseases, including cancer and dementia. The effects are mainly due to a direct impact on cell structures, the modulation of antioxidant pro-enzymes (such as superoxide dismutase, catalase and lipoxygenases), and the scavenging of hydroxyl, superoxide and peroxyl radicals. These in turn decrease protein and lipid oxidation and normalise cellular redox balance.[5] The study by Molino et al. (2018) has reported high antioxidant activity of natural extracts of tannins derived from chestnut and quebracho after *in vitro* digestion and fermentation with human microbiota. [6]

Tannins are also considered to be anti-inflammatory compounds. The reduction of reactive oxygen species is associated with a decrease in the development of inflammatory states, which contribute to tissue damage. The efficacy of hydrolysable tannins applied directly to inflamed tissues has been extensively documented *in vivo* and *in vitro* studies. [7] The mechanisms of action have been shown to improve macrophage functionality and to regulate the production of cytokines such as IL-1, IL-β2, IL-6, αTNF, etc. Tannin-containing supplements, administered orally, can influence the immune response at the local level of the gastrointestinal tract (on the mucosa) as well as express modulatory effects on the composition of the gut microbiota. However, anti-inflammatory effects at the systemic level are determined by metabolites produced by the transit of tannins through the gastrointestinal system, via hydrolysis and microbiota metabolism. In particular, much attention has recently been focused on the role of tannin metabolites produced by microbiota in silencing the immune response in inflammatory bowel and cardiovascular diseases. [7]

Tannins, isolated from plants as well as those obtained synthetically, have activity against a broad spectrum of viruses: enteroviruses (polio- and coxsackie-), caliciviruses (feline calicivirus, mouse norovirus), rotaviruses, influenza A virus, rhabdo- (vesicular stomatitis virus), paramyxoviruses (Sendai and Newcastle disease virus), human immunodeficiency virus, herpes simplex virus (HSV) (Type 1 and 2) and adenoviruses.[8] The ability of tannins to bind proteins can be harnessed to establish links to capsid proteins, specific viral enzymes required for viral replication or to newly synthesised viral proteins involved in the composition of new particles. Thus, tannins can affect different stages of viral replication, including the extracellular virions themselves, their binding to the cell, the penetration and replication process in the host, as well as the assembly of new viral particles and transport proteins, polysaccharides and viral enzymes. For example, ellagitannins are interesting HSV anti-viral agents, as their targets are virus-specific proteins, thus retaining activity against acyclovir-resistant strains of HSV types 1 and 2. [8]

**Tannins for the prevention and treatment of viral diseases.**

Antiviral drugs are limited in number, and in many cases, their use is accompanied by the presence of side effects or the formation of viral resistance, which makes the therapy partially ineffective. Therefore, the use of nature-derived remedies can contribute to find new effective therapies.

Among the plant extracts that have been studied, tannins have been found to be good antivirals. More specifically, tannins act by inhibiting the replication of different viruses. The resulting effects have been demonstrated on both coated viruses (influenza viruses A/H3N2 and A/H5N3, herpes simplex virus type 1, vesicular stomatitis virus, Sendai virus and Newcastle disease virus) [9,10] and non-enveloped viruses (poliovirus, coxsackievirus, adenovirus, rotavirus, feline calicivirus and mouse norovirus).[11]

Tannin bark extract of Hamamelis virginiana L. has been tested a different concentration for its effect against influenza A virus. The study showed that the life cycle of influenza A is inhibited in the first and, to a lesser extent, the subsequent steps. High molecular weight tannins inhibited both IAV receptor binding and neuraminidase activity, while low molecular weight tannins could inhibit neuraminidase but not haemagglutination.[12] The in vitro study by Haidari et al. (2009) has reported that punicalgin, an ellagitannin present in pomegranate extract, inhibits human influenza A (H3N2) virus RNA replication. Furthermore, the authors suggest a synergistic effect of natural products such as tannins together with the antiviral therapy Oseltamivir.[13]

In another study, replication of human, swine and duck influenza virus was counteracted *in vitro* by hydrolysable type tannins.[14]

It is well known that viral infections are accompanied by cell injury, caused by excessive generation of free radicals and consequent development of a pro-inflammatory state in the cell. Tannins also act indirectly by mitigating the effects of viral infection, thanks to their antioxidant and anti-inflammatory activity.

In the case of acute respiratory viral infections, hyperproduction of reactive oxygen species (ROS) and reactive nitrogen species (RNS) has been linked to lung tissue injury and epithelial barrier dysfunction, which in turn increases susceptibility to secondary infections, triggering a heightened inflammatory response.[15]

The high concentration of tannins present in apple extract has been shown to be effective against the development of influenza virus infection in mice: it improves survival rates, significantly decreases lipid peroxidation and also increases the oxygen radical scavenging capacity in the splenocytes. Tannins also modulated the development of influenza by elevating immune functions in mice, which may be due to their antioxidant activity directly or indirectly on immunocytes.[16]

The available information suggests that the antiviral, anti-inflammatory and antioxidant activities of tannins, widely studied in veterinary and human fields, would be a strategy to prevent and control viral respiratory diseases, as well as to diminish the more lethal effects such as excess inflammatory response. Furthermore, oxidative stress and stress-mediated complications of viral infections can be successfully countered with antioxidant prevention.

However, it has to be highlighted that the application of tannins in the prevention and treatment of viral infections has an auxiliary function, and may be particularly beneficial when used in combination with a specific therapy.

**Safety of polyphenols**

With regard to the safety of the product, no side effects were observed in humans at the doses used, taking into account the methodological limitations of the published studies.

In the context of the COVID-19 pandemic, a study is proposed to evaluate the efficacy of a polyphenolic compound based on chestnut and quebracho extract in improving the response to standard treatment.

This randomised controlled trial aims to evaluate the efficacy and safety of the tannin-based dietary supplement ARBOX in COVID-19 positive patients.

**Objectives {7}**

To evaluate the efficacy of the specific blend of chestnut and quebracho tannin extracts contained in ARBOX in SARS-CoV-2 infection.

To evaluate the safety of chestnut and quebracho tannin extracts contained in ARBOX in SARS-CoV-2 infection.

To compare baseline characteristics of COVID-19 patients vs. healthy controls.

**Trial design {8}**

Randomized, placebo-controlled, double-blind, double-blind, randomized clinical trial.

**Methods: Participants, interventions and outcomes**

**Study setting {9}**

The study and the patient collection are carried out at the Hospital de Clínicas, Buenos Aires, Argentina.

**Eligibility criteria {10}**

***Inclusion Criteria:***

Over 18 years of age hospitalised in the general inpatient ward, meeting the definition of "COVID-19 confirmed case" (https://www.argentina.gob.ar/salud/coronavirus-COVID-19/definicion-de-caso).

***Exclusion Criteria:***

- Pregnancy
- Breastfeeding
- Hypersensitivity to polyphenols
- Patients unable to receive oral medication (severe cognitive impairment, ventilator-assisted, impaired consciousness)
- Lack of consent

**Who will take informed consent? {26a}**

The investigator assesses patient’s ability to decide and extent of potential consciousness impairment based on GCS and other appropriate clinical measures. Patient with decision-making capacity will go through the standard procedure (informative interview with the investigator, written information for the patients, the possibility to ask questions, and adequate time to discuss with family and decide). If the patient wishes to participate, he/she will provide written prospective informed consent.

**Additional consent provisions for collection and use of participant data and biological specimens {26b}**

There was no additional consent.

**Interventions**

**Explanation for the choice of comparators {6b}**

There are many studies reporting the efficacy of tannins in acute viral illnesses, through modulation of the intestinal microbiota and strengthening of the immune system. The safety of tannins has also been reported. The comparison will be made on ARBOX + standard treatment against COVID-19 at the time of recruitment, vs. placebo + standard treatment.

**Intervention description {11a}**
*Treatment group:* ARBOX, a dietary supplement containing dry extract of chestnut and quebracho tannin extracts + Vitamin B12, in capsules Indunor S.A. (Table 1).

2 tablets per day for 14 days.

*Table 1Table of composition of ARBOX*

| **COMPOSITION** | **%** | **mg/Capsule** |
| --- | --- | --- |
| Polyphenol dry extract | 59.17 | 240.00 |
| Gelatine capsule | 23.57 | 95.60 |
| Corn starch | 15.73 | 63.80 |
| Silicon dioxide | 0.76 | 3.10 |
| Magnesium stearate | 0.76 | 3.10 |
| Vitamin B12 | 0.0002 | 0.0007 |
| TOTAL | 100 | 405.60 |

*Control group:* Two capsules per day, identical to the original but without the nutritional supplement (placebo) will be administered for 14 days.

Standard treatment includes: Antipyretics and antibiotic therapy, as appropriate (treatment currently recommended by the Infectious Diseases Department of the Hospital de Clínicas, which may be modified in the following days). In addition, if necessary: supplementary O2, non-invasive ventilation, antibiotic therapy.

**Criteria for discontinuing or modifying allocated interventions {11b}**

The assigned intervention will not be modified under any circumstances.

Reasons for early termination of a patient's participation include:

- Withdrawal of informed consent (subject's decision to withdraw for any reason);
- Important deviation in the process of informed consent;
- Life-threatening adverse reaction to tannin at the discretion of the investigator;
- Newly emerged pregnancy of a participant after the enrolment;

The sponsor reserves the right to discontinue the study at any time if there is a significant safety concern (e.g. grade 4 adverse reactions to tannin), or insufficient recruitment despite intensified efforts to enrol patients, or if repeated poor study documentation occurs at a site. The trial can be discontinued by the decision of the regulatory authority or ethics committee, as well.

**Strategies to improve adherence to interventions {11c}**

Patients will be contacted daily by telephone to ensure compliance with the treatment.

**Relevant concomitant care permitted or prohibited during the trial {11d}**

Patients from both groups will receive special care during the internship, which may include the use of antibiotics, corticoids, convalescent plasma and antivirals.

**Provisions for post-trial care {30}**

Post-trial care will consist in helping arrange clinical care or social services after a trial's conclusion and referral to appropriate follow-up care in the health-care sector.

**Outcomes {12}**

PRIMARY OUTCOME:

Days to discharge

SECONDARY OUTCOMES:

1) Mortality at day 28

2) Invasive ventilation requirement at Day 28

3) Percentage of CRP negativization at day 21

4) Level of inflammation parameters (erythrocyte sedimentation, CRP, ferritin, albumin). Vitamin D. Difference day 1 and 14

5) Level of pro- and anti-inflammatory cytokines. Difference day 1 and 14

6) Adverse effects: the proportion and frequency in each branch of the following symptoms during treatment should be recorded: diarrhoea at day 14.

7) Changes in faecal microbiota and intestinal permeability between day 1 and 14.

The primary objective of this study is to test the hypothesis that the administration of ARBOX results in better outcomes compared to placebo in adult patients with mild, moderate or severe confirmed COVID-19 as a supplementation of standard treatment.

The secondary objective is to investigate de efficacy and safety of ARBOX and thus to explore the capacity of ARBOX to modulate the inflammation, the production of cytokines and to regulate the composition of the intestinal microbiota, in comparison to the placebo.

**Participant timeline {13}**

Patients will be assessed for inclusion within 24 hours prior to recruitment. On the day of recruitment, patients will sign the informed consent form and will be given the assigned intervention.

At enrolment:

- Eligibility screen – inclusion and exclusion criteria
- Informed consent
- Demographic data (age, sex, race or ethnic group, BMI)
- Medical history (allergies, comorbidities and CCI, chronic medication)
- Vital signs (temperature, blood pressure, heart rate, respiratory rate)

Day 1:

- Vital signs (temperature, blood pressure, heart rate, respiratory rate)
- Administration of tannin or placebo according to the allocation to treatment arms
- Blood count and routine biochemistry (RBC, WBC, haematocrit, lymphocyte count, platelets, coagulation profile, D-dimer, LDH, troponin, pH, blood gases, sodium, potassium, chloride, glycaemia) renal and liver functions (BUN, creatinine, AST, ALT, bilirubin)
- Inflammatory markers (CRP, ferritin, erythrocyte sedimentation)
- Cytokine levels
- Faecal sample microbiota analysis

Day 1-28 / ICU discharge (whichever comes first):

- Checking adverse events and adverse reactions
- Ventilatory status (e.g. use of a mechanical ventilator or other ventilation/oxygen support)
- Administration of tannin or placebo according to the allocation to treatment arms
- Vital signs (temperature, blood pressure, heart rate, respiratory rate)

Day 14:

- Inflammatory markers (CRP, ferritin, erythrocyte sedimentation)
- Cytokine levels
- Faecal sample microbiota analysis

**Figure**. **Clinical trial schedule**

|  | **STUDY PERIOD** | | | | | |
| --- | --- | --- | --- | --- | --- | --- |
|  | **Enrolment** | **Allocation** | **Post-allocation** | **Close-out** | **Follow-up** | |
| **TIMEPOINT**** | ***-24HS*** | **0** | ***D1*** | ***D14*** | ***D21*** | ***D28*** |
| **ENROLMENT:** | | | | | | |
| **Inclusion/Exclusion criteria** | X |  |  |  |  |  |
| **Informed consent** | X |  |  |  |  |  |
| ***Randomization*** |  | X |  |  |  |  |
| **INTERVENTIONS:** | | | | | | |
| ***ARBOX*** |  |  |  |  |  |  |
| ***PLACEBO*** |  |  |  |  |  |  |
| **ASSESSMENTS:** | | | | | | |
| Demographic data | X |  |  |  |  |  |
| Medical history | X |  |  |  |  |  |
| Vital signs | X |  |  |  |  |  |
| Blood count and routine biochemistry^1^ |  |  | X |  |  |  |
| Inflammation parameters^2^ |  |  | X | X |  |  |
| Cytokines^3^ |  |  | X | X |  |  |
| Adverse effects^4^ |  |  |  | X |  |  |
| Changes in intestinal status^5^ |  |  | X | X |  |  |
| CRP negativization |  |  |  |  | X |  |
| Invasive ventilation |  |  |  |  |  | X |
| Mortality |  |  |  |  |  | X |
| Adverse events |  |  |  |  |  |  |

^1^ RBC, WBC, haematocrit, lymphocyte count, platelets, coagulation profile, renal and liver functions (BUN, creatinine, AST, ALT, bilirubin), D-dimer, LDH, troponin, pH, blood gases, sodium, potassium, chloride, glycaemia]

^2^Erythrocyte sedimentation, CRP, ferritin, albumin. Vitamin D. Difference day 1 and 5

^3^ IL-1B, IIL- 1ra, L-2, IL-3, IL- 4, IL-5, IL-6, IL-8, IL-9, IL-10, IL- 12, IL-13, IL-15, IL-17, basic FG, Eotaxin, G-CSF, GM-CSF, IFN gamma, IP-10, MCP-1, MIP 1 alfa, MIP 1 beta, PDGF- BB, RAN-TES, TNF alfa, VEGF

^4^ Assessment of the presence of diarrhoea

^5^ Diarrhoea, metagenomic analyses of faecal microbiota and assessment of intestinal permeability

**Sample size {14}**

Considering an alpha error of 5%, a power of 80% and a Relative Risk Reduction of 15%, a sample size of 70 patients per arm was estimated. All patients undergoing randomization will be evaluated.

**Recruitment {15}**

All patients meeting the criteria for a suspect case with a positive nasal swab PCR result will be evaluated for inclusion. Enrolled patients will be given the INFORMED CONSENT FORM (Annex 1), to be returned signed, and the PATIENT INFORMATION SHEET (Annex 2).

**Assignment of interventions: allocation**

**Sequence generation {16a}**

Patients will be randomized in 1:1 ratio in one of the two treatment arms, using a simple computer randomisation system. The allocation sequences will be prepared by one of the investigators independent of the study team. Allocation to the treatment arm of an individual patient will not be available to the physicians before the completion of the whole randomization process.

Following stratification factors will be applied:

Severity:

- Mild: no pneumonia
- Moderate: pneumonia + oxygen saturation>94%
- Severe: pneumonia + oxygen saturation <94%

**Concealment mechanism {16b}**

The allocation will be delivered in an opaque, sealed and numbered envelope.

**Implementation {16c}**

The allocation sequences will be prepared by one of the investigators independent of the study team. Recruitment and allocation of interventions will be done by the interventional physicians.

**Assignment of interventions: Blinding**

**Who will be blinded {17a}**

Both patients and interventional physicians will not be aware of the allocation sequence.

**Procedure for unblinding if needed {17b}**

It will not be allowed to unmask the assigned intervention.

**Data collection and management**

**Plans for assessment and collection of outcomes {18a}**

Baseline data will be collected from the medical record. Outcome data will be collected by the treating physician, daily. Adverse event data will be collected by the treating physician through the daily telephone interview with the patient. Duplication of data will be avoided through a computerized system created for this protocol, which avoids uploading duplicate data from the same participant. There will be a dedicated data entry person, who will be trained for this purpose, to upload the data into the system. Descriptions of the methods used for laboratory determinations are described in the material and methods section.

**Plans to promote participant retention and complete follow-up {18b}**

Participants will be contacted daily by telephone to ensure their compliance.

**Data management {19}**

Data will be loaded into an informatized online system, created exclusively for this study, which prevents duplication of data, by a trained person.

**Confidentiality {27}**

Participants will be identified by an ID number to maintain confidentiality. Thus, data will be shared without the affiliation data of the participants.

**Plans for collection, laboratory evaluation and storage of biological specimens for genetic or molecular analysis in this trial/future use {33}**

Biological samples of blood and fecal material will be collected. Blood samples be centrifuged and stored at -70 degrees until processing. Fecal samples will be transported in conditions suitable for their preservation to the reference laboratory for DNA extraction and then sent to the laboratories for further processing for DNA sequencing.

**Statistical methods**

**Statistical methods for primary and secondary outcomes {20a}**

Analyses will be performed based on the full analysis set, which is defined as the set of all randomized patients who received at least one treatment specified in the trial. Statistical analysis will be performed on randomly assigned treatment groups. Continuous variables will be summarized by presenting the median and interquartile range (IQR) for the total number of patients who contributed values. Categorical variables will be summarized by presenting the frequency and proportion of patients in each category. Time-to-event data will be analyzed using the Kaplan-Meier method, and the median time to event and corresponding 95% CI were calculated. Hazard ratios (HRs) with 95% CIs will be calculated using Cox proportional hazards models. Treatment effects for secondary endpoints will be assessed using odds ratios with 95% CIs. Statistical analyses will be performed with Stata software (Stata Corp., College Station, Texas, USA) version 16.1. Statistical significance will be defined using a 2-sided significance level of α = .05.

As regards microbiome sequencing, the differences in alpha diversity will be evaluated, based on the data distribution of metrics, using ANOVA and Tukey's HSD (honestly significant difference) tests for normally distributed data or Wilcoxon-Mann-Whitney with Holm-Bonferroni correction method for non-normally distributed data. To compare microbial composition between samples, beta-diversity will be measured by calculating the weighted or unweighted UniFrac distance matrix. Principal coordinates analysis (PCoA) will be applied on the distance matrices to generate bi-dimensional plots in R. The permutational analysis of variance (PERMANOVA) test will be calculated calculated using the function *adonis* in the *vegan* package  (CRAN - Package vegan. <https://cran.r-project.org/web/packages/vegan/index.html>.) and will be performed to determine whether there is a significant separation between different sample groups.

A p-value < 0.05 after False Discovery Rate (FDR) correction will be considered as statistically significant. Linear discriminant analysis (LDA) effect size (LEfSE) algorithm will be also used to discover potential bacterial biomarkers associated to COVID-19 patients. The differences in abundance will be regarded as significant when the logarithmic LDA score was higher than 2.

**Interim analyses {21b}**

An interim analysis will be performed at the completion of half of the projected patients. The decision whether or not to discontinue the trial on the basis of these results will be taken by the entire team of investigators involved.

**Methods for additional analyses (e.g. subgroup analyses) {20b}**

Pre-planned subgroup analysis will be performed regarding the primary outcome variable in the subgroups defined by the following criteria:

• Severity of disease (mild, moderate, severe)

• Age

• Sex

• BMI

• Comorbidities

**Methods in analysis to handle protocol non-adherence and any statistical methods to handle missing data {20c}**

No imputation techniques for missing data will be applied.

**Plans to give access to the full protocol, participant level-data and statistical code {31c}**

There are no plans to make the database public. Interested investigators can access it by requesting it by email to the principal investigator.

**Oversight and monitoring**

**Composition of the coordinating center and trial steering committee {5d}**

Not applicable.

**Composition of the data monitoring committee, its role and reporting structure {21a}**

Not applicable.

**Adverse event reporting and harms {22}**

Adverse event (AE) could be diseases or symptoms which occur or worsen after the enrolment of a patient in

the clinical trial. All AEs need to be documented, no matter if the investigator suspects a causal

connection to the study medication. AE will be monitored and documented from the day of

giving informed consent until the end of participation in the study.

Subjects will be instructed to report any AEs that they experience to the investigator. The investigator should actively ask about AEs.

Each AE should be described, documented in the eCRF, and evaluated to determine:

• Seriousness.

• Severity.

• Causality, i.e. relation to the study medication;

• Duration (start and end dates or whether it continues).

• Action taken (no action taken; study medication discontinued; prolongation of the ongoing hospitalization; administration of a drug etc.) AE needs to be followed until its resolution, i.e. until it subsides, stabilizes, becomes chronic, or the subject dies.

**Frequency and plans for auditing trial conduct {23}**

Not applicable.

**Plans for communicating important protocol amendments to relevant parties (e.g. trial participants, ethical committees) {25}**

All changes to the protocol were communicated to the ethics committee and the teaching and research department in writing.

**Dissemination plans {31a}**

The results will be disseminated as peer reviewed articles in indexed scientific journals.

**Trial status**

Recruitment completed.

**Abbreviations**

AE Adverse Event

ALT Alanine aminotransferase

ANOVA Analysis of variance

AST Aspartate aminotransferase

BMI Body mass index

BUN Blood urea nitrogen

CCI Charlson Comorbidity Index

CI Confidence interval

COVID-19 Coronavrus Disease 2019

eCRF (electronic) Case Report Form

CRP C-reactive protein

DNA Deoxyribonucleic acid

FDR False Discovery Rate

G-CSF Granulocyte colony-stimulating factor

GCS Glasgow coma scale

GM-CSF Granulocyte-macrophage colony-stimulating factor

HSD Honestly significant difference

HR Hazard ratio

HSD Honestly significant difference

HSV Herpex simplex virus

IAV Influenza A virus

ICU Intensive Care Unit

IFNγ Interferon gamma

IL Interleukin

IP-10 interferon-inducible protein 10

IQR Interquartile range

LDA Linear discriminant analysis

LDH Lactate dehydrogenase

LEfSE Linear discriminant analysis effect size

MCP-1 monocyte chemoattractant protein-1

MIP-1 Macrophage inflammatory protein-1

MERS-CoV Middle East Syndrome Coronavirus

PCoA Principal coordinates analysis

PCR Polymerase chain reaction

PDGF- BB Platelet-derived growth factor -BB

PERMANOVA Permutational analysis of variance

RAN-TES Regulated on activation, normal T cell expressed and secreted

RBC Red blood cells

RNA Ribonucleic acid

RNS Reactive nitrogen species

ROS Reactive oxygen species

RT-PCR Polymerase chain reaction with reverse transcription

SARS-CoV Syndrome-related Coronavirus

SCFA Short chain fatty acid

TNF-α Tumor necrosis factor alpha

VEGF Vascular endothelial growth factor

WBC White blood cells

WGS Whole genome sequencing

**Declarations**

**Consent for publication**

Not applicable.

**Availability of data and materials**

Not applicable

**Competing interests**

The authors declare that they have no competing interests.

**Funding**

﻿This work was supported by Indunor/Silvateam SA founding.

**Authors' contributions**

All authors made a substantial contribution to the design and the concept of the study. All authors read and approved the final version of this summary.

**Acknowledgements**

Not applicable

**Authors’ contributions {31b}**

All authors made a substantial contribution to the design and the concept of the study. All authors read and approved the final version of the final manuscript.

**Funding {4}**

This work was supported by Indunor/Silvateam SA founding. The funder was not involved in the design of the study and collection, analysis, and interpretation of data and in writing the manuscript.

**Availability of data and materials {29}**

Participating researchers will have access to the final data. The data will be used and disseminates as stipulated in the contractual agreement.

**Ethics approval and consent to participate {24}**

This study was approved on 12th June 2020 by the Ethics Committee of the Hospital de Clínicas Jose de San Martin, Buenos Aires University, Argentina. Written informed consent was obtained from all study participants or their legal representatives.

**Consent for publication {32}**

Not applicable

**Competing interests {28}**

The authors declare that they have no competing interests

**References**

1. Williamson G, Manach C. Bioavailability and bioefficacy of polyphenols in humans. II. Review of 93 intervention studies. Am J Clin Nutr. 2005;81 (suppl):243S-55S.

2. Sieniawska E, Baj T. Tannins [Internet]. Pharmacogn. Fundam. Appl. Strateg. Elsevier Inc.; 2017. Available from: http://dx.doi.org/10.1016/B978-0-12-802104-0.00010-X

3. Sieniawska E. Activities of tannins-From in Vitro studies to clinical trials. Nat Prod Commun. 2015;10:1877–84.

4. Khanbabaee K, van Ree T, Ree T van. Tannins: Classification and definition. Nat Prod Rep. 2001;18:641–9.

5. Molino S, Casanova NA, Rufián Henares JÁ, Fernandez Miyakawa ME. Natural Tannin Wood Extracts as a Potential Food Ingredient in the Food Industry. J Agric Food Chem. 2019;68:2836–48.

6. Molino S, Fernández-Miyakawa M, Giovando S, Rufián-Henares JÁ. Study of antioxidant capacity and metabolization of quebracho and chestnut tannins through in vitro gastrointestinal digestion-fermentation. J Funct Foods. 2018;49:188–95.

7. Kiss AK, Piwowarski JP. Ellagitannins, Gallotannins and their Metabolites- The Contribution to the Anti-Inflammatory Effect of Food Products and Medicinal Plants. Curr Med Chem. 2016;25:4946–67.

8. Vilhelmova-Ilieva N, S. Galabov A, Mileva M. Tannins as Antiviral Agents. In: Aires A, editor. Tann - Struct Prop Biol Prop Curr Knowl. IntechOpen; 2020. p. 1–13.

9. Uozaki M, Yamasaki H, Katsuyama Y, Higuchi M, Higuti T, Koyama AH. Antiviral effect of octyl gallate against DNA and RNA viruses. Antiviral Res [Internet]. 2007 [cited 2020 Apr 20];73:85–91. Available from: https://linkinghub.elsevier.com/retrieve/pii/S0166354206002336

10. Müller Kratz J, Regina Andrighetti-Fröhner C, Juliana Kolling D, César Leal P, César Cirne-Santos C, Augusto Yunes R, et al. Anti-HSV-1 and anti-HIV-1 activity of gallic acid and pentyl gallate [Internet]. Mem Inst Oswaldo Cruz, Rio Janeiro. 2008. Available from: http://www.scielo.br/pdf/mioc/v103n5/285.pdf

11. Ueda K, Kawabata R, Irie T, Nakai Y, Tohya Y. Inactivation of Pathogenic Viruses by Plant-Derived Tannins: Strong Effects of Extracts from Persimmon (Diospyros kaki) on a Broad Range of Viruses. PLoS One [Internet]. 2013 [cited 2020 Apr 20];8:55343. Available from: www.plosone.org

12. Theisen LL, Erdelmeier CAJ, Spoden GA, Boukhallouk F, Sausy A, Florin L, et al. Tannins from Hamamelis virginiana bark extract: Characterization and improvement of the antiviral efficacy against influenza a virus and human papillomavirus. PLoS One. 2014;9:1–14.

13. Haidari M, Ali M, Ward Casscells S, Madjid M. Pomegranate (Punica granatum) purified polyphenol extract inhibits influenza virus and has a synergistic effect with oseltamivir. Phytomedicine [Internet]. 2009 [cited 2020 Apr 20];16:1127–36. Available from: https://linkinghub.elsevier.com/retrieve/pii/S0944711309001652

14. Saha RK, Takahashi T, Kurebayashi Y, Fukushima K, Minami A, Kinbara N, et al. Antiviral effect of strictinin on influenza virus replication. Antiviral Res [Internet]. 2010 [cited 2020 Apr 20];88:10–8. Available from: https://linkinghub.elsevier.com/retrieve/pii/S0166354210006418

15. De Marco F. Oxidative stress and HPV carcinogenesis. Viruses. 2013;5:708–31.

16. He RR, Wang M, Wang CZ, Chen BT, Lu CN, Yao XS, et al. Protective effect of apple polyphenols against stress-provoked influenza viral infection in restraint mice. J Agric Food Chem. 2011;59:3730–7.
